# Using 8-Hydroxy-2′-Deoxiguanosine (8-OHdG) as a Reliable Biomarker for Assessing Periodontal Disease Associated with Diabetes

**DOI:** 10.3390/ijms25031425

**Published:** 2024-01-24

**Authors:** Ancuta Goriuc, Karina-Alexandra Cojocaru, Ionut Luchian, Ramona-Garbriela Ursu, Oana Butnaru, Liliana Foia

**Affiliations:** 1 Department of Biochemistry, Faculty of Dental Medicine, “Grigore T. Popa” University of Medicine and Pharmacy, 16 Universității Street, 700115 Iasi, Romania; ancuta.goriuc@umfiasi.ro (A.G.); karina-alexandra.cojocaru@d.umfiasi.ro (K.-A.C.); georgeta.foia@umfiasi.ro (L.F.); 2 Department of Periodontology, Faculty of Dental Medicine, “Grigore T. Popa” University of Medicine and Pharmacy, 16 Universității Street, 700115 Iasi, Romania; 3 Department of Preventive Medicine and Interdisciplinarity (IX)—Microbiology, Faculty of Medicine, “Grigore T. Popa” University of Medicine and Pharmacy, 700115 Iasi, Romania; 4 Department of Biophysics, Faculty of Dental Medicine, “Grigore T. Popa” University of Medicine and Pharmacy, 16 Universității Street, 700115 Iasi, Romania; oana.maria.butnaru@umfiasi.ro

**Keywords:** 8-hydroxy deoxy guanosine, oxidative stress, periodontitis, periodontal disease, diabetes mellitus, biomarkers

## Abstract

In recent years, research has shown that oxidative stress plays a significant role in chronic inflammatory conditions. The alteration of the oxidant/antioxidant balance leads to the appearance of free radicals, important molecules involved in both diabetes mellitus and periodontal disease. Diabetes is considered to be one of the major risk factors of periodontal disease and the inflammation characterizing this condition is associated with oxidative stress, implicitly resulting in oxidative damage to DNA. 8-Hydroxydeoxyguanosine (8-OHdG) is the most common stable product of oxidative DNA damage caused by reactive oxygen species, and its levels have been reported to increase in body fluids and tissues during inflammatory conditions. 8-OHdG emerges as a pivotal biomarker for assessing oxidative DNA damage, demonstrating its relevance across diverse health conditions, including neurodegenerative disorders, cancers, inflammatory conditions, and periodontal disease. Continued research in this field is crucial for developing more precise treatments and understanding the detailed link between oxidative stress and the progression of periodontitis. The use of the 8-OHdG biomarker in assessing and managing chronic periodontitis is an area of increased interest in dental research, with the potential to provide crucial information for diagnosis and treatment.

## 1. Introduction

Oxidative stress, a change that causes significant damage to the body, denotes the disruption of the delicate physiological equilibrium between oxidants and antioxidants, with the former being favored. Chemically speaking, free radicals are atoms, ions, or molecules with unpaired electrons on their outer orbital, giving them exceptional chemical reactivity and a pronounced tendency to attack other molecules to obtain the necessary electron for stabilization. By generating reactive species that subsequently capture additional electrons in a cascade effect, radicals participate in chain reactions characterized by radical addition and substitution. Their negative impact manifests in destabilizing cells by attacking various cellular components and molecules (including lipids and proteins in the cellular membrane, as well as mitochondrial DNA), causing mutations or even cellular death [1].

Although most free radicals have a short lifespan, there are highly stable radicals that pose the most danger to the human body. If free radicals are the cause of many diseases, then it means that they are everywhere, and, indeed, that is the case. Where there is life, there are free radicals—both allies and adversaries. Free radicals cause severe damage, but, paradoxically, we cannot live without them. Free radicals and related species have well-defined roles in the inflammatory process [2]. The balance between the oxidative action of free radicals and the level of antioxidants in an organism represents the mechanism responsible for maintaining a state of health. In many pathological conditions, there is an acceleration in the formation of reactive oxygen species (ROS), resulting in an imbalance between oxidant factors and protective antioxidant systems [3]. Thus, the involvement of free radicals in over 100 conditions is explained, including periodontal disease, by triggering lesions at the protein, lipid, and DNA levels [4].

Periodontitis represents a highly studied inflammatory disease characterized by the gradual destruction of the supporting tissues of the teeth, ultimately leading to tooth loss if not properly treated [5]. Periodontal disease is a pathological process mediated by the interaction between dysbiotic microbial populations and aberrant immune responses at the level of periodontal tissues [6]. Over time, this mechanism leads to the destruction of periodontal tissues and a continuous positive feedback response of proteolysis, inflammation, and an increase in periodontal pathogens. Numerous studies in recent years have led to a better understanding of the pathogenesis of periodontal disease, involving the host’s immune response, the importance of the oral microbiome, and risk factors [7,8,9]. Consequently, the goal of periodontal disease therapy has recently shifted towards restoring oral microbiota homeostasis while maintaining a harmonious balance at the periodontal level [10]. Other studies have focused on the association between diabetes mellitus and periodontal disease, with the latter being considered the sixth complication of diabetes [11]. To date, the mechanisms by which diabetes mellitus exacerbates periodontal disease are not fully understood. An evaluation of oxidative stress biomarkers could be useful in screening for periodontal disease associated with diabetes mellitus, and one of these biomarkers resulting from DNA damage is 8-OHdG [12,13,14].

The damage caused to DNA by ROS has sparked profound interest in the medical world due to their involvement in various pathological conditions. 8-OHdG is an oxidized nucleoside excreted in the body as a reparative consequence of DNA [4]. It is a marker involved in the pathogenesis of malignant, inflammatory, autoimmune, and diabetic diseases. A correlation has been observed between the salivary levels of 8-OHdG and periodontal microbiota, indicating the effectiveness of this biomarker in determining the periodontal status [15].

The objective of the current narrative review is focused on establishing if 8-OHdG may represent a reliable a valuable marker for early detection, diagnosis, follow-up, and management of periodontal disease in diabetic patients for improving their prognosis.

Using the following keywords: 8-hydroxy deoxy guanosine; oxidative stress; periodontitis; periodontal disease; diabetes mellitus; and biomarkers, two specialists investigated, independently, the main scientific databases PubMed and Web of Science. In order to avoid the risk of bias, the investigators were instructed to include both positive and negative papers regarding the effectiveness of the 8-OHdG marker. Only the publications that were selected by both independent investigators were considered reliable and included in this narrative review. Through this process, 106 publications were selected.

## 2. Periodontal Disease and Diabetes: An Interconnected Relationship through Oxidative Stress

### 2.1. Diabetes Mellitus and Periodontal Disease

The term “diabetes mellitus” designates a spectrum of disorders characterized by elevated blood glucose levels. Although periodontal disease is initiated by biofilm, diabetes is well recognized as a contributory risk factor in this pathological process. The interrelation between periodontal disease and systemic conditions becomes apparent through shared risk factors, particularly the subgingival dental biofilm acting as a continual reservoir for proinflammatory cytokines, such as interleukin-1β (IL-1β), tumor necrosis factor-alpha (TNFα), and prostaglandin E2 (PGE2). The well-vascularized nature of the inflamed periodontium in individuals with periodontal disease results in a sustained release of various substances, including cytokines, potentially modifying diverse physiological states and pathological processes [16].

Taylor and Borgnakke identified periodontal disease as a potential factor contributing to suboptimal metabolic control in individuals with diabetes mellitus. Diabetes is associated with an elevated incidence and progression of periodontitis. Furthermore, the presence of periodontal infection correlates with poorer glycemic control in individuals with diabetes. Several studies have indicated an association between periodontal disease and an increased risk of complications in individuals with diabetes. The management of periodontal infection in diabetes patients is not only vital for maintaining oral health but may also play a crucial role in establishing and sustaining glycemic control, potentially delaying the onset or progression of diabetes-related complications [17].

### 2.2. Oxidative Stress in Diabetes and Periodontal Disease

Oxidative stress, defined as an imbalance between the formation of free radicals and antioxidant defense, has been recognized as an important factor in the occurrence of serious pathologies, such as atherosclerosis, diabetes, rheumatoid arthritis, neurodegenerative diseases, stroke, heart attack, and cancer, and it is believed to underlie the aging process [18,19].

ROS, including hydrogen peroxide (H_2_O_2_), hydroxyl radical (OH^•^), and superoxide anion radical (O_2_^•−^), are normal cellular metabolism products, acting as a defense mechanism against diseases associated with phagocytic infiltration. This process represents a host defense mechanism against bacterial pathogens [20,21].

However, ROS overproduction leads to the oxidation of DNA, lipids, and proteins, contributing to tissue damage [22,23]. Bacterial pathogens stimulate host cells, leading to the release of pro-inflammatory cytokines (e.g., IL-1β and TNFα) as part of the immune response. These cytokines recruit polymorphonuclear leukocytes to the site of infection. Stimulated by bacterial antigens (e.g., lipopolysaccharides), these leukocytes produce proteolytic enzymes such as elastase and trigger a series of oxidation reactions catalyzed by nicotinamide adenine dinucleotide phosphate oxidase (NADPH oxidase). Consequently, ROS are released into the extracellular environment, resulting in host tissue damage [24,25].

Antioxidants, such as vitamin C, vitamin E, and reduced glutathione, can reduce the damage caused by ROS. Another enzyme that aids in clearing reactive oxygen species is superoxide dismutase (SOD) [26,27].

Recent research has suggested that oxidative stress is also involved in the etiopathogenesis of periodontal disease. Halliwell and Whiteman proposed some criteria to establish the direct influence of ROS on disease occurrence [28]:

➢ ROS or oxidative damage must always be present at the site of tissue injury.
➢ The appearance of free radicals must coincide with the moment of tissue injury.
➢ The direct application of ROS within a specific time frame and concentration should lead to similar oxidative tissue damage.
➢ Preventing ROS formation or removing them from the site of injury should reduce tissue damage.

At the periodontal level, ROS production occurs primarily in polymorphonuclear cells and results in non-selective cell damage, cellular membrane lipids, molecules, proteins, DNA, and matrix components [2]. ROS can lead to the degradation of proteoglycans, including hyaluronic acid and collagen, as some studies have shown [29].

At the level of type II collagen, in vitro studies have shown that ROS can induce fragmentation, polymerization, and oxidative modification, making the collagen molecule more susceptible to proteolysis [30,31]. Periodontal studies have indicated increased local collagen degradation, evidenced by the presence of its metabolites in gingival fluid. During periodontal disease, connective tissue destruction by ROS is highlighted by the presence of neutrophil infiltrates in response to bacterial invasion [32,33].

Although the direct effects of ROS on bone resorption have not been studied in patients with periodontal involvement, research suggests that certain oxygen species (O_2_^•−^ and H_2_O_2_) produce and activate osteoclasts, suggesting a possible involvement of ROS in bone resorption in periodontal disease [34,35,36].

Another significant mechanism of ROS involvement in periodontal disease involves the activation of cytokines (TNFα and Interleukin-1), chemokines (Interleukin-8), and cell adhesion molecules, leading to accelerated cellular apoptosis through DNA damage caused by nitroxyl radicals (NO). However, chronic inflammatory periodontal disease, accompanied by periodontal tissue destruction, results in increased ROS production. Studies have revealed increased levels of free radicals such as H_2_O_2_ and NO in patients with periodontal involvement [37,38].

**Figure 1:**
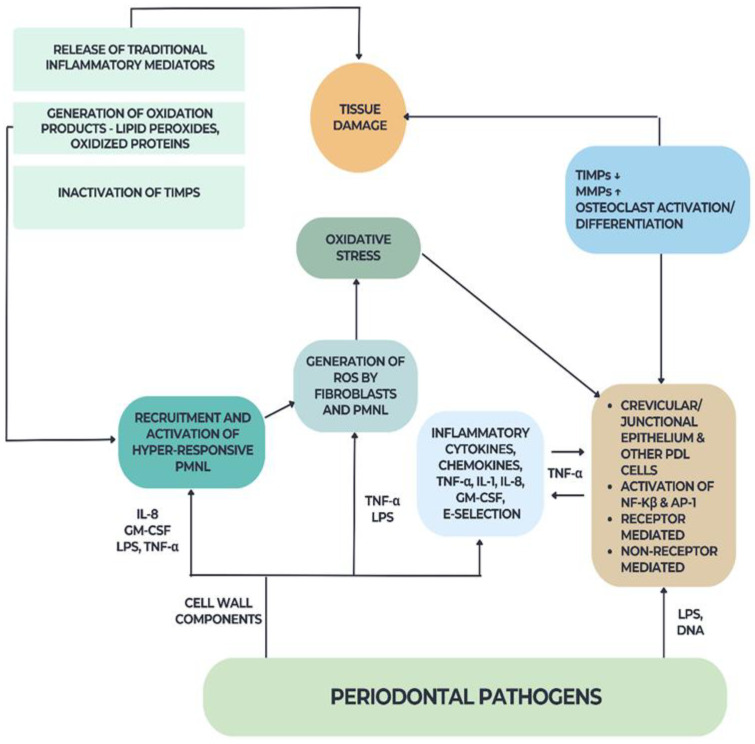
Effect of oxidative stress on the periodontium.

ROS generated during periodontal disease do not have immediate effects locally but are disseminated into the blood, leading to the oxidation of various biomolecules in the bloodstream and subsequently causing systemic oxidative stress with multiple negative effects [14] (Figure 1).

The presence of the bacterium *Treponema denticola* in the periodontal area can lead to the metabolism of reduced glutathione, altering the oxidant/antioxidant balance in the periodontium, as indicated by Chu and colleagues in their study [39]. Additionally, the presence of *Peptostreptococcus micros* in the periodontal area results in the production of hydrogen sulfide, which can strongly inhibit the activity of superoxide dismutase in human gingival fibroblasts. The reality is that bacteria in the oral cavity and periodontal pockets consume antioxidants, thereby suppressing the elimination of ROS. Consequently, defense against oral cavity bacteria decreases, allowing ROS to pass from periodontal tissues into the systemic circulation [40,41].

Periodontal disease and diabetes are closely interlinked conditions. Research has shown that individuals with diabetes are at a higher risk of developing periodontal disease due to various factors, including compromised immune responses, impaired wound healing, and increased susceptibility to infections (Table 1).

**Figure 2:**
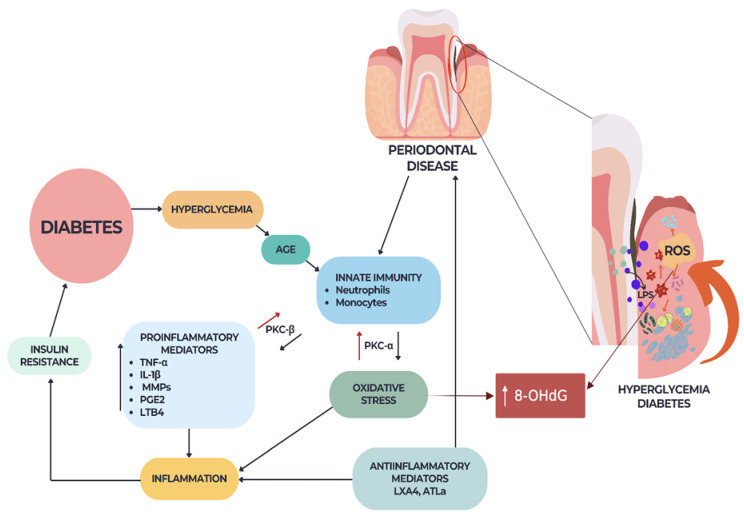
Periodontal disease–diabetes–ROS interrelation.

Oxidative stress, characterized by an imbalance between free radicals and antioxidants, plays a crucial role in the development and progression of both periodontal disease and diabetes (Figure 2). In periodontal disease, the presence of increased levels of oxidative stress leads to tissue damage and inflammation in the gums and the supporting structures of the teeth. This further exacerbates the condition, causing a breakdown of the periodontal tissues and eventual tooth loss [18].

Oxidative stress amplifies the number of free radicals generated during normal cellular processes, and the biochemical pathways strictly related to hyperglycemia (glucose auto-oxidation or prostaglandin synthesis) can increase free radical production [22]. The elevated glucose levels characteristic of diabetes affect endothelial cells, potentially leading to increased ROS. Recent research has led to two hypotheses: firstly, diabetes mellitus causes increased oxidative stress in periodontal tissues, exacerbating the disease, and, secondly, periodontal disease may worsen the destruction of pancreatic β cells [52,53].

Monea et al. confirmed a strong interconnection between oxidative stress, periodontal disease, and diabetes mellitus [45]. A study conducted by Vincent et al. aimed to compare antioxidant capacity and oxidative stress among four groups of patients: one with generalized periodontal disease without diabetes, another with both periodontal disease and diabetes, a third group with diabetes but no periodontal disease, and a final group of systemically and periodontally healthy patients. The results showed that systemically and periodontally healthy patients presented the highest levels of total antioxidant capacity. The oxidative stress index significantly differed statistically among the four groups, with the highest values observed in the group of patients with diabetes and periodontal disease [49].

Based on data from the literature, it can be concluded that poor metabolic control of diabetes mellitus may be associated with higher levels of oxidative stress and more severe periodontal lesions. An increase in glutathione peroxidase and glutathione reductase enzymes was observed in patients with good metabolic control of diabetes compared to those with poor metabolic control [46].

Similarly, in diabetes patients, the increased oxidative stress contributes to complications by damaging cells, impairing insulin sensitivity, and causing inflammation, thus aggravating the diabetic condition.

Moreover, there is a bidirectional relationship between periodontal disease and diabetes. Poorly controlled diabetes can worsen periodontal disease, and, conversely, untreated periodontal disease can negatively impact glycemic control in diabetic individuals, making it more challenging to manage blood sugar levels [54].

Therefore, managing both periodontal disease and diabetes involves controlling oxidative stress through lifestyle changes, proper oral hygiene practices, regular dental check-ups, and maintaining optimal blood sugar levels. Addressing one condition can positively impact the other, emphasizing the importance of comprehensive care in managing these interconnected health issues [55].

## 3. 8-OHdG—An Overview

### 3.1. General Aspects

**Figure 3:**
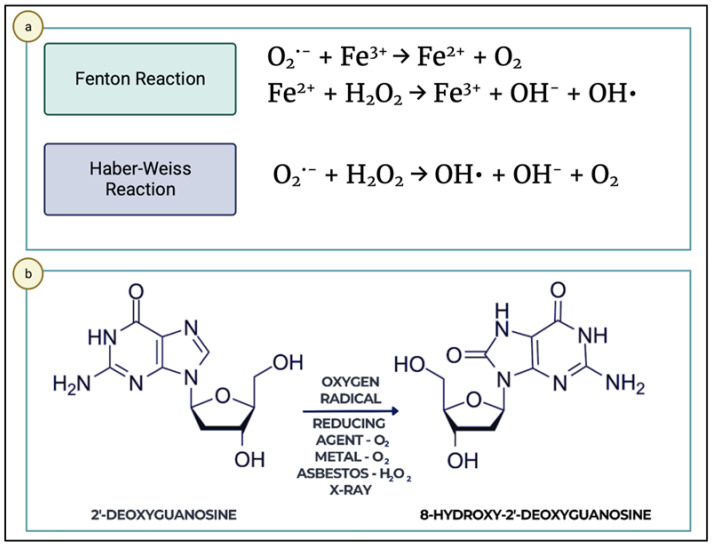
(**a**,**b**) Hydroxyl radical and formation of its scavengers and their involvement in health and disease.

Although oxygen free radicals are involved in numerous physiological biological processes, such as cellular signaling, apoptosis, and gene expression, at higher concentrations, ROS can cause irreversible damage in the body. In living organisms, two major types of ROS are produced, namely, O2^•−^ and OH^•^, with the latter being implicated in causing damage to basic biomolecules (proteins, membrane lipids, and DNA). The Haber–Weiss reaction, in which O_2_^•−^ and OH^•^ are generated in the presence of H_2_O_2_ and iron ions, has the potential to occur at the cellular level, representing a plausible origin of oxidative stress. The reaction is very slow and involves an initial step of reducing ferric ions to ferrous ions but is catalyzed by iron, and the second step is represented by the Fenton reaction (Figure 3) [56].

8-OHdG is a marker that can be measured in bodily fluids, and its reliability makes it a standard marker for DNA modifications induced by oxidation. Guanine is a nitrogenous base with a relatively low oxidation potential compared to other DNA bases, yet it is frequently attacked by various reactive species. This oxidation product of guanine can lead to the occurrence of transversion mutations, such as guanine binding to thymine or guanine binding to adenine, with detrimental consequences. Therefore, modifications to guanine through oxidation, halogenation, alkylation, etc., are among the most abundant DNA lesions upon oxidative exposure and can lead to a multitude of lethal damages [57].

The double-stranded DNA in the human mitochondrial genome comprises 16,569 base pairs. Each mitochondrion contains between two and ten copies of mitochondrial DNA (mtDNA), which encode 13 genes essential for oxidative phosphorylation (OXPHOS), 2 ribosomal RNA genes, and the transfer RNA (tRNA) genes necessary for mtDNA expression. Additionally, in each cell, there are several hundred to several thousand mitochondria. ROS and other free radicals are continuously generated near the inner mitochondrial membranes. Human mtDNA is naked and located close to these inner membranes, thus is not protected by histones. Consequently, mtDNA replicates faster than nuclear DNA without efficient DNA repair systems or corrections [58].

Recent research has shown the involvement of mtDNA mutations, such as deletions or point mutations, in the development of pathological conditions such as degenerative diseases or the aging process. For instance, a “common 5 bp deletion” seems to appear in various tissues among elderly individuals [4,59,60].

### 3.2. 8-OHdG in Pathology

Currently, 8-OHdG stands as a key marker used for evaluating oxidative DNA damage, with implications in diverse health conditions, such as neurodegenerative disorders; cancers; and other chronic inflammatory conditions, including periodontal disease. Moreover, the observed oxidative stress in conditions such as inflammatory bowel disease and type 2 diabetes mellitus (T2DM) actively stimulates inflammation [2,14,61,62].

A meta-analysis study revealed elevated 8-OHdG levels in individuals with cardiovascular disease (CVD) compared to controls. Additional pertinent insights have highlighted the independence of the association between 8-OHdG levels and CVD from factors such as diabetes, hyperlipidemia, and body mass index [63]. Elevated levels of 8-OHdG have also been shown to have associations with clinical outcomes in strokes, revealing a significant correlation with atherosclerotic plaque types and vascular recurrence in stroke patients [64]. The coexistence of oxidative stress and inflammation is frequently observed as interconnected consequences in chronic kidney disease, with serum 8-OHdG serving as a predictor for an elevated risk of all-cause mortality across various estimated glomerular filtration rate ranges. Importantly, this predictive association remains independent of inflammation [65].

In the field of neurodegenerative disorders, serum 8-OHdG emerges as a promising blood biomarker for Alzheimer’s disease, indicating its potential value in stratifying patients for treatment options due to increased levels [64]. Furthermore, elevated concentrations of 8-OHdG have been documented in the parietal cortex and various peripheral compartments, including serum and leukocytes, among individuals diagnosed with Huntington’s disease [66]. Numerous investigations have indicated elevated levels of 8-OHdG in the substantia nigra, caudate nucleus, and various brain regions among individuals with Parkinson’s disease (PD) compared to controls. While dopaminergic neurons are widely recognized as the most susceptible cells in PD, several studies have proposed the involvement of other neuronal cells, lymphocytes, and visceral organs in the disease process. Consequently, these cells may represent potential sources of increased 8-OHdG [67].

Additionally, elevated 8-OHdG levels are intricately linked to mutations that trigger carcinogenesis, with meta-analytical findings establishing a connection between a high 8-OHdG concentration in leukocyte DNA, advanced age, and poor prognosis in serous ovarian cancer patients compared to controls [64]. Another study suggests a significant involvement of oxidative stress in colorectal cancer (CRC) development, and the substantial elevation of urinary 8-OHdG emerges as a promising fluid biomarker for estimating risk, providing early warnings, and facilitating the detection of CRC [68]. Nevertheless, in the case of breast cancer, a low expression of 8-OHdG is linked to a poorer prognosis, potentially attributed to antioxidant mechanisms within breast cancer tissues [69].

The role of oxidative damage in aging is further underscored, supported by research exploring 8-OHdG as a marker for age-related damage accumulation. Animal models have contributed to this exploration, revealing increased levels of 8-OHdG in tissue and urine samples [64].

Recent research has shown an increased level of the marker 8-OHdG in the saliva of pregnant patients, as well as in the saliva of smokers. Furthermore, it seems that the salivary level of this marker is influenced by systemic conditions, such as diabetes, obesity, and hyperlipidemia, independent of periodontal disease. Thus, considering the results of previous studies, we can conclude that the serum levels of this marker depend on systemic conditions, while local, salivary levels in gingival fluid are more closely linked to periodontal status [55].

#### 3.2.1. 8-OHdG and Diabetes Mellitus

Elevated blood glucose levels and the T2DM are intricately associated with the induction of oxidative stress [70]. H_2_O_2_, OH^•^, and O_2_^•−^ are continuously generated as byproducts of metabolic reactions. Various cellular components, such as mitochondria (recognized as the primary producers of ROS), peroxisomes (responsible for fatty acid degradation), and the cytochrome P450 system (part of the mixed-function oxidation system), contribute to the ongoing release of ROS and play a pivotal role in pathophysiological mechanisms [71].

In the context of hyperglycemia, there is a notable escalation in ROS levels, thereby initiating alterations in cellular homeostasis and contributing to the formation of lesions at this level [4]. The production of O_2_^•−^ impedes the translocation efficiency of glucose transporter 4 from intracellular compartments, resulting in an elevation of blood glucose levels [72,73]. Hyperglycemia induces the overproduction of ROS, activating glucose autoxidation and increasing the formation of advanced glycosylation end products in diabetic individuals [13,74]. Concurrently, alterations at the OXPHOS level and a reduction in NADH oxidoreductase contribute to the development of insulin resistance [75].

Diabetic complications, which substantially contribute to the mortality of individuals with diabetes, are well-established in their association with oxidative damage [76,77]. The increased levels of oxidized DNA, proteins, and lipids observed in various tissues of individuals with diabetes provide compelling evidence for the presence of oxidative stress, further implicating its role in the genesis and progression of diabetic complications [78].

Typically, nuclear and mtDNA serve as primary sites for oxidative damage [79]. Among the various purine and pyrimidine bases, guanine is particularly susceptible to oxidation. The oxidative process involves the addition of a hydroxyl group to the eighth position of the guanine molecule, resulting in the oxidatively modified product known as 8-OHdG. The conversion of deoxyguanosine to 8-OHdG is associated with increased mtDNA deletions. Suzuki et al. demonstrated that both 8-OHdG and the mtDNA deletion of 4977 bp serve as valuable biomarkers for assessing oxidative stress in diabetic patients [80].

The measurement of serum or urinary 8-OHdG can be conducted utilizing various laboratory techniques, including immunological assays such as an enzyme-linked immunosorbent assay, immunofluorescence, and AKLIDES, or analytical methodologies such as high-performance liquid chromatography and liquid chromatography mass spectrometry [13].

Individuals with T2DM generally present significantly higher concentrations of 8-OHdG in their serum than control subjects. Furthermore, urinary 8-OHdG has been documented as a sensitive biomarker for oxidative stress. Negishi et al. reported a positive correlation between hemoglobin A1c (HbA1c) and 24 h urinary 8-OHdG excretions [81]. Moreover, in their study, Leinonen et al. illustrated a substantial increase in the total 24 h urinary excretion of 8-OHdG among non-insulin-dependent diabetic patients when compared with control subjects [82].

Al-Aubaidy and Jelinek demonstrated that elevated 8-OHdG levels in both prediabetic and diabetic cohorts exceeded control values, implying 8-OHdG’s potential as an early disease marker with increased sensitivity compared to cholesterol, malondialdehyde, and erythrocyte-reduced glutathione levels. This observation carries clinical significance, as 8-OHdG levels strongly indicate oxidative-stress-related DNA damage within blood vessels and tissues, consequently elevating the risk of cardiovascular disease [83].

In a study by Dincer et al., there was a reduction in the levels of reduced glutathione, a cellular antioxidant, among individuals with T2DM. This decline in reduced glutathione levels was associated with an increased incidence of oxidative DNA damage [84]. Furthermore, Xu et al. revealed an elevated urinary excretion of 8-OHdG in individuals with diabetic nephropathy, particularly among those with macroalbuminuria [85]. Increased levels of urinary 8-OHdG and leukocyte DNA were observed in individuals with diabetes. Moreover, the concentration of urinary 8-OHdG in these patients demonstrated a correlation with the severity of diabetic complications [13].

These investigations collectively highlight the intricate connections between oxidative stress, biomarkers, and diabetic complications, offering valuable insights into potential diagnostic and therapeutic avenues. Elevated blood glucose levels in T2DM induce oxidative stress, generating ROS and causing cellular disruptions. This oxidative damage, observed in various tissues, is implicated in diabetic complications. Notably, 8-OHdG, a marker of DNA damage, serves as a potential indicator, with its measurement in serum or urine aiding diagnostic efforts [83].

#### 3.2.2. 8-OHdG and Chronic Versus Aggressive Periodontitis

The microorganisms responsible for periodontal tissue destruction lead to the generation of ROS, thereby causing oxidative damage to DNA. A study conducted by Sugano and colleagues regarding the salivary assessment of the bacterium *Streptococcus anginosus* and the 8-OHdG marker in patients with chronic periodontitis showed a higher level of 8-OHdG in those with the presence of this bacterium in saliva than in those without the bacterium. Additionally, a four-month treatment for chronic periodontitis resulted in a significant decrease in the salivary levels of 8-OHdG compared to initial levels [86].

Similarly, another study involving the salivary detection of bacteria, such as *Aggregatibacter actinomycetemcomitans*, *Porphyromonas gingivalis*, and *Tannerella forsythia*, and 8-OHdG in patients with chronic periodontitis revealed an association between high salivary levels of 8-OHdG and the presence of *Porphyromonas gingivalis*, levels which significantly decreased after periodontal treatment [87].

In their study, Suwamoto and his collaborators showed a positive correlation between *Porphyromonas gingivalis* and the level of the marker 8-OHdG, with a decrease in this marker observed after periodontal treatment. The level of 8-OHdG was not correlated with clinical data, age, or smoking status [88]. Results from another study conducted on patients with chronic periodontitis also showed a positive correlation between the level of the 8-OHdG marker and the presence of *Porphyromonas gingivalis* bacteria. The development of periodontal disease involves the attachment of bacterial agents to the periodontal tissue, leading to the recruitment of polymorphonuclear leukocytes (PMNs) and the production of ROS by these cells. Thus, the level of 8-OHdG increases in the saliva of these patients [89].

Chronic periodontitis is considered an infectious condition characterized by inflammation and progressive damage to the tooth-supporting tissues, leading to gradual attachment loss and eventual tooth loss. In the case of patients with chronic periodontal involvement, oxidative stress plays a crucial role. Oxidative stress can amplify the inflammatory response, subsequently triggering chain reactions that lead to pronounced tissue destruction. One of the important markers for evaluating oxidative stress in these patients is 8-OHdG in saliva and gingival fluid [90]. Studies have shown elevated levels of this marker in patients with chronic periodontitis. Additionally, measuring this marker can provide insights into the prognosis of the disease [61].

Higher salivary levels of this marker in saliva and gingival fluid correspond to a less favorable prognosis. Previous research has also shown higher levels of 8-OHdG in smokers than in non-smokers. This reliable marker of oxidative DNA damage can be useful for monitoring treatment by measuring it in saliva or gingival fluid before and after treatment [15,91].

Aggressive periodontitis is a particular form of periodontal disease characterized by an intense inflammatory response and rapid loss of tooth attachment.

Studies investigating the role of 8-OHdG in aggressive periodontitis suggest a connection between this marker of oxidative stress and the progression of this more aggressive form of periodontal condition. A comparative study between patients with chronic periodontitis and those with aggressive periodontitis showed significantly higher concentrations of 8-OHdG in the gingival fluid of the latter group [92].

Another comparative study between periodontally healthy patients, those with chronic periodontitis, and those with aggressive periodontitis aimed to identify interleukin-1 and 8-OHdG in the saliva of these patients. The results showed the highest concentrations of these markers in patients with periodontitis [93]. A study focused on the correlation between the salivary levels of 8-OHdG and the depth of periodontal pockets (PD) in patients with aggressive periodontitis concluded that the salivary levels of 8-OHdG correlate with the depth of periodontal pockets. Patients with periodontal pockets between 6 and 9 mm showed higher values of 8-OHdG than those with pockets between 4 and 5 mm [94].

Zamora-Perez et al. conducted a study on cells exfoliated from the oral mucosa, evaluating DNA damage in these cells by counting micronuclei and nuclear alterations. The 8-OHdG marker was identified in saliva as a marker of oxidative stress. Patients with aggressive periodontitis showed a higher number of nuclear anomalies and micronuclei than the group of patients with chronic periodontitis, showing a positive correlation with the 8-OHdG marker [95].

Based on the above studies, it can be concluded that there is a positive correlation between the level of oxidative stress, nuclear anomalies, inflammatory cytokine levels, and the 8-OHdG marker concerning the degree of periodontal involvement in both aggressive and chronic periodontitis. Research continues to investigate the specific role of 8-OHdG in chronic and aggressive periodontitis to develop therapeutic strategies and better understand the connection between oxidative stress and the progression of this condition. The use of 8-OHdG as a marker in the evaluation and management of chronic periodontitis represents an area of increased interest in dental research [3].

These studies shed light on a significant link between the marker 8-OHdG and the progression of periodontitis, both in its aggressive and chronic forms. The higher concentrations of 8-OHdG in gingival fluid in patients with aggressive periodontitis than in those with chronic periodontitis indicate a higher level of DNA damage caused by oxidative stress in the more aggressive form of the disease [96,97].

Furthermore, the relationship between the salivary levels of 8-OHdG and the depth of periodontal pockets in patients with aggressive periodontitis suggests a correlation between this marker and the severity of periodontal involvement. Moreover, studies conducted on exfoliated cells from the oral mucosa have highlighted the connection between increased levels of 8-OHdG and nuclear anomalies, suggesting an association between oxidative stress and DNA alterations in aggressive periodontitis [95].

Given the importance of periodontal disease as a health issue, we thought to analyse the research studies published in the last 10 years, regarding association of 8-OHdG biomarker in pathogenesis and development of periodontal disease. Using PubMed data base, we identified nine articles published between 2014and 2024. All these studies used different versions of the ELISA assay for the detection of 8-OHdG in saliva, with one single exception which tested serum also [98]. A research team from Turkey used a second assay, beside ELISA: liquid chromatography with tandem mass spectrometry [99]. The identified studies made comparison between healthy patients and different selected groups of patients, as: pregnant women, diabetic patients, menopausal groups and premenopausal groups, and patients with acute coronary syndrome. Using a specific statistical analysis, the authors found an important association between salivary levels of 8-OHdG and diabetic patients, menopausal and premenopausal women, patients with acute coronary syndrome. A research team from Spain identified a good correlation between this biomarker and known etiologic agents of periodontal disease: *Porphyromona gingivalis, Aggregatibacter actinomycetemcomitans, Treponema denticola*, and *Tannerella forsythia* [100,101,102]. As a continuation, a research team from the Universidad de Valencia, Valencia, Spain, published a meta-analysis, 3 years later. The authors concluded that 8-OHdG could be considered as a powerful marker of periodontal disease, given the more increased concentration of salivary 8-OHdG in periodontal disease, in comparison with healthy control groups [103] (Table 2).

In experienced hands, ELISA can be a useful tool in evaluation a biomarker involvement on periodontal disease development and progression. Nowadays, there is a need for more sensitive, specific, and clinically validated laboratory assays, as NGS, MALDI TOF, for a better understanding of salivary 8-OHdG biomarker and its associations with known bacterial etiologic agents of periodontal diseases. More patients should be tested for this biomarker, from different geographic areas, for getting the whole information about this health topic.

## 4. Conclusions and Perspectives

8-OHdG emerges as a pivotal and versatile biomarker for assessing oxidative DNA damage, with significant relevance across different health conditions such as neurodegenerative disorders, cancers, diabetes, other inflammatory processes, or periodontal disease.

We can state that this paper highlights the intricate relationship between diabetes and periodontitis incidence and progression, as well as the association with oxidative DNA damage.

The management of periodontal infection in diabetic patients is crucial not only for oral healthcare but also for better glycemic control. Furthermore, the studies analyzed indicate a positive correlation between the level of oxidative stress marker- 8-OHdG, inflammatory cytokines, and periodontal pathogens.

Thus, the use of the promising 8-OHdG marker in assessing and managing periodontitis is an area of increased interest for dental research and requires future studies. Continuous improvement of the laboratory techniques used to correctly assess this marker for periodontal disease in diabetic patients can significantly contribute to developing future treatment protocols and also to improving clinical guidelines for diabetes.

## Figures and Tables

**Table 1:** The associations between oxidative stress, diabetes and periodontal disease.

Author, Year	Design of the Study	Oral Oxidative Stress Biomarker	Results	Conclusion
Akalın et al., 2008 [42]	Cross-sectional	Gingival: Superoxide dismutase	HbA1c, glucose, and triglyceride levels were higher in diabetic groups; There were correlations between periodontal parameters and superoxide dismutase values.	Superoxide dismutase values increased in diabetic patients.
Jung et al., 2013 [43]	Cross-sectional	Gingival: inducible nitric oxide synthase	Inducible nitric oxide synthases and tissue inhibitors of metalloproteinase were significantly higher in diabetic and periodontitis patients as compared to healthy group.	Inducible nitric oxide synthase values increased in diabetes and chronic periodontitis group.
Pendyala et al., 2013 [44]	Cross-sectional	Saliva: Total antioxidant capacity	Total antioxidant capacity is proportional to the inflammation.	Total antioxidant was lower in diabetic patients with periodontal disease.
Monea et al., 2014 [45]	Cross-sectional	Biopsy specimen (dental-periodontal unit in the posterior region of dental arches): Malondialdehyde Glutathione	Histological alterations in diabetic patients were present in gingival mucosa (epithelium and lamina propria).	Malondialdehyde levels was higher in diabetic tissues. Glutathione values was significantly lower in diabetic tissues.
Arana et al., 2017 [46]	Cross-sectional	Saliva: glutathione peroxidase, glutathione reductase, reduced glutathione, oxidized glutathione.	The diabetic patients with good metabolic control showed a significant increase in glutathione peroxidase and glutathione reductase activity.	Elevated levels of oxidative stress in the saliva of individuals with diabetes are correlated with poorer metabolic control, and a deteriorated state of periodontal health.
Muthuraj et al., 2017 [47]	Prospective study	Gingival crevicular fluid: 8-OHdG	Levels of 8-OHdG and HbA1c in periodontal patients with diabetes showed a greater reduction after scaling and root planing.	8-OHdG values increased in diabetic and periodontitis patients.
Koregol et al., 2018 [48]	Cross-sectional	Saliva: 8-isoprostane	A statistically significant difference was observed in the concentrations of 8-isoprostane among the various groups.	8-isoprostane values decreased in diabetic and periodontitis group.
Vincent et al., 2018 [49]	Cross-sectional	Gingival crevicular fluid: Total antioxidant capacity	The clinical parameters (plaque index, gingival index, probing pocket depth) showed the statistically significant difference between the groups.	Total antioxidant capacity was higher in healthy group.
Shee et al., 2020 [50]	Cross-sectional	Saliva: Malondialdehyde	Malondialdehyde levels showed an exponential rise with the inclusion of diabetes and TBSCH, while no such increase was noted in relation to periodontitis.	Malondialdehyde values increased in diabetic and periodontitis group.
Shashikumar et al., 2022 [51]	Prospective study	Saliva: Total antioxidant capacity	Total antioxidant level was lower in diabetic and periodontitis group after 3 months therapy with Morinda citrifolia L. mouthwash.	Total antioxidant level was lower in diabetic and periodontitis group.

**Table 2:** The associations between 8-OHdG, diabetes, and periodontal disease.

Author, Year	Patients/Method of 8-OHdG Detection	Results	Clinical Importance
Altıngöz SM et al., 2021 [104]	periodontitis patients +/− diabetes 8-OHdG ELISA	the combination of 8-OHdG for diabetes patients yielded highest AUCs	salivary 8-OHdG levels were significantly higher in periodontitis compared to controls
Kemer Doğan ES et al., 2017 [100]	menopausal groups and premenopausal 8-OHdG ELISA	8-OHdG levels were higher in menopausal groups than in premenopausal ones	salivary 8-OHdG levels may be used as an indicator of the relationship between periodontal disease
Nguyen TT et al., 2017 [101]	patients with acute coronary syndrome (ACS) 8-OHdG ELISA	salivary levels of 8-OHdG, were significantly higher in the chronic periodontitis ACS, ACS, and CP	salivary 8-OHdG had a strong potential to be utilized in chronic periodontitis investigation
Önder C et al., 2017 [98]	healthy individuals and diagnosed with CP. saliva and serum 8-OHdG ELISA	8-OHdG levels in the saliva were significantly higher in the CP group in comparison with the control group	salivary 8-OHdG levels may be used as indicators for severity of periodontal disease
Shin MS et al., 2016 [102]	residents participated in both dental and medical examinations 8-OHdG ELISA	TZhe association of salivary 8-OHdG with severe periodontitis increased from OR of 2.40 to OR of 4.10 for drinking and 3.14 for smoking	the association between salivary level of 8-OHdG and severe periodontitis was significant salivary 8-OHdG could be a useful marker for severe periodontitis.
Kurgan Ş et al., 2015 [99]	healthy patients with CP and healthy individuals salivary 8-OHdG ELISA liquid chromatography with tandem mass spectrometry analysis 8-OHdG	levels of 8-OHdG were significantly higher in the chronic periodontitis group compared to the control group pretreatment values of LC-MS/MS 8-OHdG also showed a positive correlation with 8-OHdG measured by ELISA	salivary levels of 8-OHdG measured by LC-MS/MS and ELISA were significantly higher in the chronic periodontitis group compared to the control group. 8-OHdG correlates well with the clinical parameters of periodontal disease. LC-MS/MS could be used to determine the lower concentrations of 8-OHdG that are not in the detection range of the ELISA.
Villa-Correa YA et al., 2015 [105]	patients with untreated chronic periodontitis and healthy controls salivary levels of 8-OHdG ELISA	8-OHdG salivary levels were significantly increased in the CP group in comparison to healthy group	increased salivary levels of 8-OHdG may be strong/independent prognostic indicators of the amount and extent of oxidative stress-induced periodontal breakdown
Almerich-Silla JM et al., 2015 [106]	individuals with periodontal problems 8-OHdG ELISA porphyromona gingivalis, aggregatibacter actinomycetemcomitans, treponema denticola, and tannerella forsythia was detected by PCR	oxidative stress levels were significantly higher in the periodontal disease group	the 8-OHdG levels were increased in the presence of all bacterial types. determination of these levels and periodontal bacteria could be a potent tool for controlling periodontal disease development.

## Data Availability

Not applicable.
